# 1,3-Bis(2,6-diisopropyl­phen­yl)-1*H*-imidazol-3-ium bromide dichloro­methane disolvate

**DOI:** 10.1107/S1600536812022246

**Published:** 2012-05-23

**Authors:** Matthias Berger, Norbert Auner, Tanja Sinke, Michael Bolte

**Affiliations:** a Institut für Anorganische und Analytische Chemie, Goethe-Universität Frankfurt, Max-von-Laue-Strasse 7, 60438 Frankfurt am Main, Germany

## Abstract

In the title compound, C_27_H_37_N_2_^+^·Br^−^·2CH_2_Cl_2_, both the cation and the anion are located on a crystallographic mirror plane. Both of the dichloro­methane solvent mol­ecules show a disorder across a mirror plane over two equally occupied positions. In the crystal, the cations are connnected to the bromide ions *via* C—H⋯Br hydrogen bonds.

## Related literature

For the preparation of imidazolium salts, see: Arduengo *et al.* (1995 ▶, 1999 ▶); Hinter­mann (2007 ▶). For structures with the same cation but different anions, see: Stasch *et al.* (2004 ▶); Blue *et al.* (2006 ▶); Berger *et al.* (2012 ▶). For compounds with the 1,3-bis-(2,6-diisopropyl­phen­yl)imidazolium unit, see: Ikhile *et al.* (2010 ▶); Giffin *et al.* (2010 ▶).
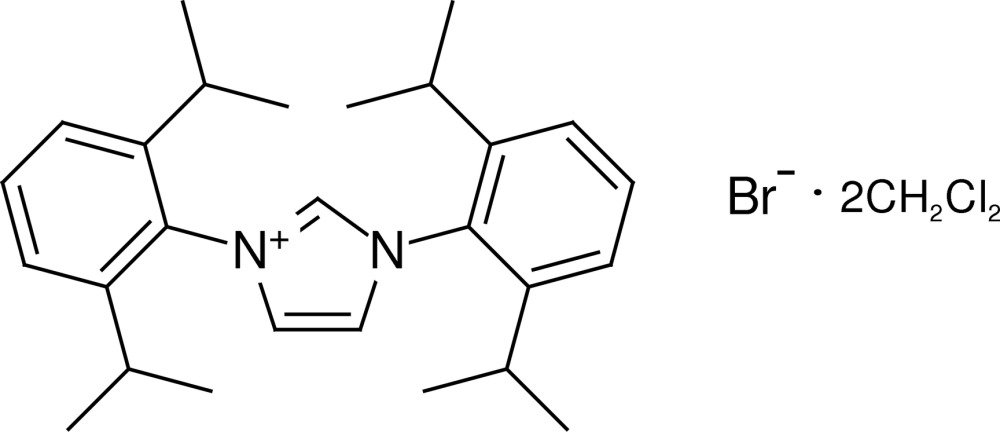


## Experimental

### 

#### Crystal data

C_27_H_37_N_2_^+^·Br^−^·2CH_2_Cl_2_
*M* *_r_* = 639.35
Monoclinic, 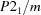

*a* = 9.1874 (8) Å
*b* = 16.5165 (12) Å
*c* = 11.030 (1) Å
β = 102.332 (7)°
*V* = 1635.1 (2) Å^3^
*Z* = 2
Mo *K*α radiation
μ = 1.60 mm^−1^
*T* = 173 K
0.52 × 0.28 × 0.24 mm

#### Data collection

Stoe IPDS II two-circle diffractometer
Absorption correction: multi-scan (*MULABS*; Spek, 2009 ▶; Blessing, 1995 ▶) *T*_min_ = 0.489, *T*_max_ = 0.700
20988 measured reflections
3200 independent reflections
2867 reflections with *I* > 2σ(*I*)
*R*_int_ = 0.084

#### Refinement

*R*[*F* ^2^ > 2σ(*F* ^2^)] = 0.038
*wR*(*F* ^2^) = 0.090
*S* = 1.03
3200 reflections
197 parameters
H-atom parameters constrained
Δρ_max_ = 0.43 e Å^−3^
Δρ_min_ = −0.36 e Å^−3^

### 

Data collection: *X-AREA* (Stoe & Cie, 2001 ▶); cell refinement: *X-AREA*; data reduction: *X-AREA*; program(s) used to solve structure: *SHELXS97* (Sheldrick, 2008 ▶); program(s) used to refine structure: *SHELXL97* (Sheldrick, 2008 ▶); molecular graphics: *XP* in *SHELXTL *(Sheldrick, 2008 ▶); software used to prepare material for publication: *SHELXL97* .

## Supplementary Material

Crystal structure: contains datablock(s) I, global. DOI: 10.1107/S1600536812022246/ng5272sup1.cif

Structure factors: contains datablock(s) I. DOI: 10.1107/S1600536812022246/ng5272Isup2.hkl

Supplementary material file. DOI: 10.1107/S1600536812022246/ng5272Isup3.cml

Additional supplementary materials:  crystallographic information; 3D view; checkCIF report

## Figures and Tables

**Table 1:** Hydrogen-bond geometry (Å, °)

*D*—H⋯*A*	*D*—H	H⋯*A*	*D*⋯*A*	*D*—H⋯*A*
C1—H1⋯Br1	0.95	2.59	3.538 (3)	175

**Fig. 1.**
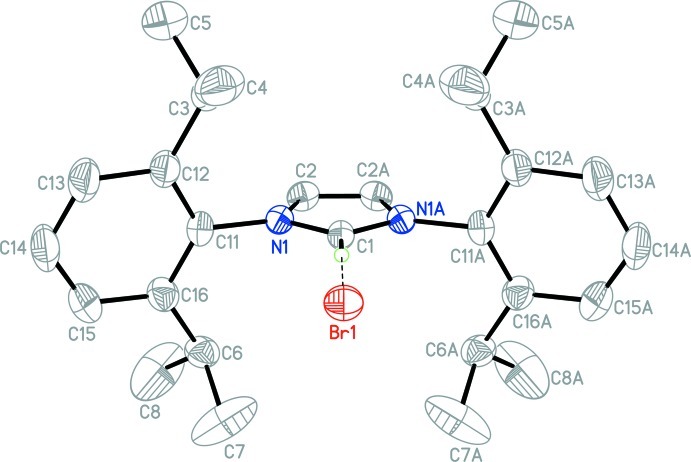
A perspective view of the title compound, showing the atom-numbering scheme. Displacement ellipsoids are drawn at the 50% probability level. Hydrogen bonds are shown as dashed lines. H atoms not involved in hydrogen bonding and dichloromethane molecules are omitted for clarity. Atoms labelled with suffix A were generated by the symmetry operator x, -y + 1/2, z.

## supplementary crystallographic information

### Comment

Imidazolium salts are precursors for the synthesis of N-heterocyclic carbenes (NHC) and can be prepared according to Arduengo *et al.* (1995, 1999) and Hintermann (2007). Deprotonation by strong bases gives the free stable NHC, which is widely used as ligands.

The title compound crystallizes with discrete cations, anions and solvent dichloromethane molecules. Both cations and anions are located on a crystallographic mirror plane. Both dichloromethane molecules show a disorder across a mirror plane over two equally occupied positions. The Br anions are connnected to the cations *via* C—H···Br hydrogen bonds. Structures with the same cation, but with different anions and solvent molecules, have been determined by Stasch *et al.* (2004), Blue *et al.* (2006) and Berger *et al.* (2012). For compounds with 1,3-bis-(2,6-diisopropylphenyl)imidazolium unit, see: Ikhile *et al.* (2010) and Giffin *et al.* (2010).

### Experimental

1,3-Bis(2,6-di-isopropylphenyl)1*H*-imidazol-3-ium bromide chloroform disolvate was prepared by reacting 167 mg of 1,3-bis(2,6-diisopropylphenyl)-1,3-dihydro-2*H*-imidazol-2-ylidene with 115 mg of Si_2_Br_6_ in deuterated dichloromethane. After two weeks at 253 K colorless needles of the title compound crystallized in the NMR-Tube.

### Refinement

H atoms were refined using a riding model, with C—H ranging from 0.95 Å to 1.00 Å and with *U*_iso_(H) = 1.2*U*_eq_(C) or *U*_iso_(H) = 1.5*U*_eq_(C_methyl_).

### Crystal data

**Table:**

C_27_H_37_N_2_^+^·Br^−^·2CH_2_Cl_2_	*F*(000) = 664
*M**_r_* = 639.35	*D*_x_ = 1.299 Mg m^−^^3^
Monoclinic, *P*2_1_/*m*	Mo *K*α radiation, λ = 0.71073 Å
Hall symbol: -P 2yb	Cell parameters from 19135 reflections
*a* = 9.1874 (8) Å	θ = 3.4–26.0°
*b* = 16.5165 (12) Å	µ = 1.60 mm^−^^1^
*c* = 11.030 (1) Å	*T* = 173 K
β = 102.332 (7)°	Plate, colourless
*V* = 1635.1 (2) Å^3^	0.52 × 0.28 × 0.24 mm
*Z* = 2	

### Data collection

**Table:**

Stoe IPDS II two-circle diffractometer	3200 independent reflections
Radiation source: Genix 3D IµS microfocus X-ray source	2867 reflections with *I* > 2σ(*I*)
Genix 3D multilayer optics monochromator	*R*_int_ = 0.084
ω scans	θ_max_ = 25.7°, θ_min_ = 3.4°
Absorption correction: multi-scan (*MULABS*; Spek, 2009; Blessing, 1995)	*h* = −11→11
*T*_min_ = 0.489, *T*_max_ = 0.700	*k* = −19→20
20988 measured reflections	*l* = −13→13

### Refinement

**Table:**

Refinement on *F*^2^	Primary atom site location: structure-invariant direct methods
Least-squares matrix: full	Secondary atom site location: difference Fourier map
*R*[*F*^2^ > 2σ(*F*^2^)] = 0.038	Hydrogen site location: inferred from neighbouring sites
*wR*(*F*^2^) = 0.090	H-atom parameters constrained
*S* = 1.03	*w* = 1/[σ^2^(*F*_o_^2^) + (0.041*P*)^2^ + 0.9357*P*] where *P* = (*F*_o_^2^ + 2*F*_c_^2^)/3
3200 reflections	(Δ/σ)_max_ < 0.001
197 parameters	Δρ_max_ = 0.43 e Å^−^^3^
0 restraints	Δρ_min_ = −0.36 e Å^−^^3^

### Special details

**Table:**

Experimental. ;
Geometry. All e.s.d.'s (except the e.s.d. in the dihedral angle between two l.s. planes) are estimated using the full covariance matrix. The cell e.s.d.'s are taken into account individually in the estimation of e.s.d.'s in distances, angles and torsion angles; correlations between e.s.d.'s in cell parameters are only used when they are defined by crystal symmetry. An approximate (isotropic) treatment of cell e.s.d.'s is used for estimating e.s.d.'s involving l.s. planes.
Refinement. Refinement of *F*^2^ against ALL reflections. The weighted *R*-factor *wR* and goodness of fit *S* are based on *F*^2^, conventional *R*-factors *R* are based on *F*, with *F* set to zero for negative *F*^2^. The threshold expression of *F*^2^ > σ(*F*^2^) is used only for calculating *R*-factors(gt) *etc*. and is not relevant to the choice of reflections for refinement. *R*-factors based on *F*^2^ are statistically about twice as large as those based on *F*, and *R*- factors based on ALL data will be even larger.

### Fractional atomic coordinates and isotropic or equivalent isotropic displacement parameters (Å^2^)

**Table:**

	*x*	*y*	*z*	*U*_iso_*/*U*_eq_	Occ. (<1)
N1	0.29660 (18)	0.18481 (10)	0.41051 (15)	0.0255 (4)	
C1	0.2134 (3)	0.2500	0.4158 (3)	0.0241 (6)	
H1	0.1119	0.2500	0.4222	0.029*	
C2	0.4378 (2)	0.20927 (14)	0.4033 (2)	0.0313 (5)	
H2	0.5197	0.1751	0.3992	0.038*	
C3	0.1307 (3)	0.11525 (15)	0.1796 (2)	0.0440 (6)	
H3	0.1846	0.1682	0.1925	0.053*	
C4	−0.0359 (4)	0.1327 (2)	0.1415 (3)	0.0594 (8)	
H4A	−0.0567	0.1626	0.0629	0.089*	
H4B	−0.0669	0.1652	0.2059	0.089*	
H4C	−0.0911	0.0815	0.1311	0.089*	
C5	0.1838 (3)	0.0696 (2)	0.0773 (3)	0.0545 (7)	
H5A	0.1607	0.1012	0.0004	0.082*	
H5B	0.1333	0.0171	0.0640	0.082*	
H5C	0.2917	0.0611	0.1017	0.082*	
C6	0.3631 (3)	0.09778 (16)	0.6435 (2)	0.0394 (5)	
H6	0.4138	0.1470	0.6197	0.047*	
C7	0.2619 (4)	0.1250 (3)	0.7253 (4)	0.0934 (15)	
H7A	0.1868	0.1621	0.6791	0.140*	
H7B	0.3203	0.1529	0.7980	0.140*	
H7C	0.2123	0.0779	0.7523	0.140*	
C8	0.4824 (5)	0.0423 (3)	0.7119 (4)	0.1023 (17)	
H8A	0.5472	0.0256	0.6565	0.153*	
H8B	0.4361	−0.0057	0.7401	0.153*	
H8C	0.5415	0.0707	0.7838	0.153*	
C11	0.2443 (2)	0.10184 (13)	0.4124 (2)	0.0317 (5)	
C12	0.1641 (3)	0.06934 (14)	0.3013 (2)	0.0382 (5)	
C13	0.1118 (4)	−0.00958 (16)	0.3072 (3)	0.0531 (7)	
H13	0.0559	−0.0343	0.2342	0.064*	
C14	0.1396 (4)	−0.05222 (16)	0.4166 (3)	0.0578 (8)	
H14	0.1019	−0.1057	0.4183	0.069*	
C15	0.2209 (3)	−0.01857 (16)	0.5234 (3)	0.0500 (7)	
H15	0.2401	−0.0494	0.5978	0.060*	
C16	0.2761 (3)	0.06008 (14)	0.5248 (2)	0.0366 (5)	
Br1	−0.17082 (3)	0.2500	0.41902 (3)	0.03239 (11)	
C9	0.6207 (8)	0.2781 (5)	0.1008 (6)	0.083 (4)	0.50
H9A	0.6687	0.2735	0.1899	0.100*	0.309 (13)
H9B	0.6987	0.2657	0.0540	0.100*	0.309 (13)
H9C	0.6825	0.2561	0.0452	0.100*	0.191 (13)
H9D	0.6666	0.2661	0.1886	0.100*	0.191 (13)
Cl1	0.5757 (3)	0.3808 (3)	0.0739 (3)	0.1306 (12)	0.50
Cl2	0.4398 (9)	0.2500	0.0600 (7)	0.091 (3)	0.38 (3)
Cl2'	0.4863 (16)	0.1966 (13)	0.0681 (6)	0.129 (7)	0.309 (13)
C10	0.7893 (8)	0.2231 (4)	0.7366 (6)	0.0643 (18)	0.50
H10A	0.7776	0.2363	0.6475	0.077*	0.50
H10B	0.7760	0.1639	0.7438	0.077*	0.50
Cl3	0.96814 (15)	0.2500	0.81560 (13)	0.0802 (4)	
Cl4	0.6535 (2)	0.27308 (11)	0.79502 (16)	0.0803 (7)	0.50

### Atomic displacement parameters (Å^2^)

**Table:**

	*U*^11^	*U*^22^	*U*^33^	*U*^12^	*U*^13^	*U*^23^
N1	0.0245 (9)	0.0254 (8)	0.0255 (8)	0.0012 (7)	0.0030 (7)	−0.0009 (7)
C1	0.0239 (14)	0.0225 (14)	0.0246 (14)	0.000	0.0022 (11)	0.000
C2	0.0247 (10)	0.0374 (11)	0.0322 (11)	0.0053 (9)	0.0074 (8)	−0.0019 (9)
C3	0.0606 (16)	0.0333 (12)	0.0331 (12)	−0.0058 (12)	−0.0012 (11)	−0.0042 (10)
C4	0.074 (2)	0.0622 (19)	0.0383 (14)	0.0213 (16)	0.0033 (13)	−0.0042 (13)
C5	0.0549 (17)	0.0630 (18)	0.0446 (15)	0.0006 (15)	0.0085 (13)	−0.0039 (14)
C6	0.0428 (13)	0.0418 (13)	0.0325 (12)	0.0040 (11)	0.0055 (10)	0.0051 (10)
C7	0.057 (2)	0.142 (4)	0.081 (3)	0.000 (2)	0.0153 (19)	−0.065 (3)
C8	0.100 (3)	0.100 (3)	0.080 (3)	0.056 (3)	−0.042 (2)	−0.025 (2)
C11	0.0341 (12)	0.0226 (10)	0.0381 (12)	0.0013 (9)	0.0073 (9)	−0.0009 (9)
C12	0.0456 (14)	0.0276 (11)	0.0384 (12)	−0.0020 (10)	0.0025 (10)	−0.0018 (10)
C13	0.071 (2)	0.0321 (13)	0.0501 (16)	−0.0120 (13)	0.0003 (14)	−0.0050 (12)
C14	0.081 (2)	0.0268 (13)	0.0620 (18)	−0.0124 (13)	0.0074 (16)	0.0036 (12)
C15	0.0673 (19)	0.0345 (13)	0.0484 (15)	−0.0004 (13)	0.0128 (14)	0.0118 (11)
C16	0.0396 (13)	0.0321 (12)	0.0381 (12)	0.0050 (10)	0.0082 (10)	0.0048 (10)
Br1	0.02843 (17)	0.03733 (18)	0.03321 (18)	0.000	0.01058 (12)	0.000
C9	0.056 (3)	0.148 (12)	0.041 (3)	−0.028 (4)	−0.003 (2)	−0.009 (4)
Cl1	0.0784 (17)	0.170 (3)	0.129 (2)	0.0489 (19)	−0.0110 (16)	−0.020 (2)
Cl2	0.068 (3)	0.144 (9)	0.063 (3)	0.000	0.019 (2)	0.000
Cl2'	0.094 (7)	0.234 (17)	0.063 (2)	−0.100 (10)	0.024 (3)	−0.035 (5)
C10	0.084 (4)	0.064 (4)	0.056 (3)	−0.011 (3)	0.039 (3)	−0.015 (3)
Cl3	0.0666 (8)	0.1076 (11)	0.0723 (8)	0.000	0.0278 (6)	0.000
Cl4	0.0721 (10)	0.099 (2)	0.0690 (9)	0.0362 (10)	0.0134 (8)	−0.0046 (9)

### Geometric parameters (Å, º)

**Table:**

N1—C1	1.329 (2)	C11—C12	1.395 (3)
N1—C2	1.377 (3)	C12—C13	1.396 (4)
N1—C11	1.454 (3)	C13—C14	1.373 (4)
C1—N1^i^	1.329 (2)	C13—H13	0.9500
C1—H1	0.9500	C14—C15	1.371 (4)
C2—C2^i^	1.346 (5)	C14—H14	0.9500
C2—H2	0.9500	C15—C16	1.393 (4)
C3—C12	1.515 (3)	C15—H15	0.9500
C3—C5	1.521 (4)	C9—Cl2	1.691 (10)
C3—C4	1.525 (4)	C9—Cl1	1.757 (10)
C3—H3	1.0000	C9—Cl2'	1.810 (11)
C4—H4A	0.9800	C9—H9A	0.9900
C4—H4B	0.9800	C9—H9B	0.9900
C4—H4C	0.9800	C9—H9C	0.9900
C5—H5A	0.9800	C9—H9D	0.9900
C5—H5B	0.9800	Cl1—Cl2'^i^	1.51 (3)
C5—H5C	0.9800	Cl2—C9^i^	1.691 (10)
C6—C7	1.496 (4)	Cl2'—C9^i^	1.280 (11)
C6—C8	1.503 (4)	Cl2'—Cl1^i^	1.51 (3)
C6—C16	1.514 (3)	Cl2'—Cl2'^i^	1.76 (4)
C6—H6	1.0000	C10—Cl4	1.731 (6)
C7—H7A	0.9800	C10—Cl3	1.745 (7)
C7—H7B	0.9800	C10—H10A	0.9900
C7—H7C	0.9800	C10—H10B	0.9900
C8—H8A	0.9800	Cl3—C10^i^	1.745 (7)
C8—H8B	0.9800	Cl4—Cl4^i^	0.763 (4)
C8—H8C	0.9800	Cl4—C10^i^	1.523 (6)
C11—C16	1.394 (3)		
			
C1—N1—C2	108.82 (18)	H8A—C8—H8C	109.5
C1—N1—C11	124.62 (18)	H8B—C8—H8C	109.5
C2—N1—C11	126.56 (18)	C16—C11—C12	124.2 (2)
N1^i^—C1—N1	108.2 (3)	C16—C11—N1	118.2 (2)
N1^i^—C1—H1	125.9	C12—C11—N1	117.7 (2)
N1—C1—H1	125.9	C11—C12—C13	116.2 (2)
C2^i^—C2—N1	107.06 (12)	C11—C12—C3	123.7 (2)
C2^i^—C2—H2	126.5	C13—C12—C3	120.1 (2)
N1—C2—H2	126.5	C14—C13—C12	121.2 (3)
C12—C3—C5	111.9 (2)	C14—C13—H13	119.4
C12—C3—C4	109.9 (2)	C12—C13—H13	119.4
C5—C3—C4	110.6 (2)	C15—C14—C13	120.8 (2)
C12—C3—H3	108.1	C15—C14—H14	119.6
C5—C3—H3	108.1	C13—C14—H14	119.6
C4—C3—H3	108.1	C14—C15—C16	121.2 (2)
C3—C4—H4A	109.5	C14—C15—H15	119.4
C3—C4—H4B	109.5	C16—C15—H15	119.4
H4A—C4—H4B	109.5	C15—C16—C11	116.4 (2)
C3—C4—H4C	109.5	C15—C16—C6	121.1 (2)
H4A—C4—H4C	109.5	C11—C16—C6	122.5 (2)
H4B—C4—H4C	109.5	Cl2—C9—Cl1	92.2 (4)
C3—C5—H5A	109.5	Cl2—C9—H9A	116.6
C3—C5—H5B	109.5	Cl1—C9—H9A	106.3
H5A—C5—H5B	109.5	Cl2'—C9—H9A	106.3
C3—C5—H5C	109.5	Cl2—C9—H9B	125.7
H5A—C5—H5C	109.5	Cl1—C9—H9B	106.3
H5B—C5—H5C	109.5	Cl2'—C9—H9B	106.3
C7—C6—C8	111.2 (3)	H9A—C9—H9B	106.4
C7—C6—C16	111.3 (2)	Cl2—C9—H9C	113.3
C8—C6—C16	112.1 (2)	Cl1—C9—H9C	113.3
C7—C6—H6	107.3	Cl2—C9—H9D	113.3
C8—C6—H6	107.3	Cl1—C9—H9D	113.3
C16—C6—H6	107.3	Cl2'—C9—H9D	100.2
C6—C7—H7A	109.5	H9C—C9—H9D	110.6
C6—C7—H7B	109.5	Cl2'^i^—Cl1—C9	45.3 (3)
H7A—C7—H7B	109.5	Cl1^i^—Cl2'—C9	106.2 (9)
C6—C7—H7C	109.5	Cl4—C10—Cl3	111.7 (3)
H7A—C7—H7C	109.5	Cl4—C10—H10A	109.3
H7B—C7—H7C	109.5	Cl3—C10—H10A	109.3
C6—C8—H8A	109.5	Cl4—C10—H10B	109.3
C6—C8—H8B	109.5	Cl3—C10—H10B	109.3
H8A—C8—H8B	109.5	H10A—C10—H10B	107.9
C6—C8—H8C	109.5		
			
C2—N1—C1—N1^i^	0.8 (3)	C12—C11—C16—C15	−0.9 (4)
C11—N1—C1—N1^i^	−179.28 (14)	N1—C11—C16—C15	178.5 (2)
C1—N1—C2—C2^i^	−0.50 (18)	C12—C11—C16—C6	−179.8 (2)
C11—N1—C2—C2^i^	179.60 (16)	N1—C11—C16—C6	−0.4 (3)
C1—N1—C11—C16	−98.5 (3)	C7—C6—C16—C15	−77.5 (4)
C2—N1—C11—C16	81.4 (3)	C8—C6—C16—C15	47.7 (4)
C1—N1—C11—C12	81.0 (3)	C7—C6—C16—C11	101.3 (3)
C2—N1—C11—C12	−99.1 (3)	C8—C6—C16—C11	−133.5 (3)
C16—C11—C12—C13	1.2 (4)	Cl2—C9—Cl1—Cl2'^i^	2.7 (5)
N1—C11—C12—C13	−178.1 (2)	Cl2'—C9—Cl1—Cl2'^i^	7.6 (4)
C16—C11—C12—C3	−179.8 (2)	Cl1—C9—Cl2—C9^i^	174.0 (2)
N1—C11—C12—C3	0.8 (4)	Cl2'—C9—Cl2—C9^i^	1.6 (6)
C5—C3—C12—C11	124.9 (3)	Cl2—C9—Cl2'—C9^i^	−177.7 (8)
C4—C3—C12—C11	−111.8 (3)	Cl1—C9—Cl2'—C9^i^	173.1 (2)
C5—C3—C12—C13	−56.2 (4)	Cl2—C9—Cl2'—Cl1^i^	175.3 (9)
C4—C3—C12—C13	67.1 (3)	Cl1—C9—Cl2'—Cl1^i^	166.1 (5)
C11—C12—C13—C14	−0.5 (4)	Cl2—C9—Cl2'—Cl2'^i^	2.3 (8)
C3—C12—C13—C14	−179.5 (3)	Cl1—C9—Cl2'—Cl2'^i^	−6.9 (2)
C12—C13—C14—C15	−0.6 (5)	Cl4—C10—Cl3—C10^i^	−50.6 (3)
C13—C14—C15—C16	0.9 (5)	Cl3—C10—Cl4—Cl4^i^	−121.8 (4)
C14—C15—C16—C11	−0.2 (4)	Cl3—C10—Cl4—C10^i^	58.2 (4)
C14—C15—C16—C6	178.7 (3)		

Symmetry code: (i) *x*, −*y*+1/2, *z*.

### Hydrogen-bond geometry (Å, º)

**Table:**

*D*—H···*A*	*D*—H	H···*A*	*D*···*A*	*D*—H···*A*
C1—H1···Br1	0.95	2.59	3.538 (3)	175
